# Altered spontaneous neural activity in the precuneus, middle and superior frontal gyri, and hippocampus in college students with subclinical depression

**DOI:** 10.1186/s12888-021-03292-1

**Published:** 2021-06-01

**Authors:** Bo Zhang, Shouliang Qi, Shuang Liu, Xiaoya Liu, Xinhua Wei, Dong Ming

**Affiliations:** 1 Department of Biomedical Engineering, Lab of Neural Engineering & Rehabilitation, College of Precision Instruments and Optoelectronics Engineering, Tianjin University, Tianjin, China; 2 College of Medicine and Biological Information Engineering, Northeastern University, Shenyang, China; 3 Tianjin International Joint Research Center for Neural Engineering, Academy of Medical Engineering and Translational Medicine, Tianjin University, No.92 Weijin Road, Nankai District, Tianjin, 300072 China; 4 Department of Radiology, Guangzhou First People’s Hospital, School of Medicine, South China University of Technology, Guangzhou, China

**Keywords:** Subclinical depression, Major depressive disorder, ALFF, ReHo, Resting-state fMRI

## Abstract

**Background:**

Subclinical depression (ScD) is a prevalent condition associated with relatively mild depressive states, and it poses a high risk of developing into major depressive disorder (MDD). However, the neural pathology of ScD is still largely unknown. Identifying the spontaneous neural activity involved in ScD may help clarify risk factors for MDD and explore treatment strategies for mild stages of depression.

**Methods:**

A total of 34 ScD subjects and 40 age-, sex-, and education-matched healthy controls were screened from 1105 college students. The amplitude of low-frequency fluctuation (ALFF) and regional homogeneity (ReHo) of resting-state fMRI were calculated to reveal neural activity. Strict statistical strategies, including Gaussian random field (GRF), false discovery rate (FDR), and permutation test (PT) with threshold-free cluster enhancement (TFCE), were conducted. Based on the altered ALFF and ReHo, resting-state functional connectivity (RSFC) was further analyzed using a seed-based approach.

**Results:**

The right precuneus and left middle frontal gyrus (MFG) both showed significantly increased ALFF and ReHo in ScD subjects. Moreover, the left hippocampus and superior frontal gyrus (SFG) showed decreased ALFF and increased ReHo, respectively. In addition, ScD subjects showed increased RSFC between MFG and hippocampus compared to healthy controls, and significant positive correlation was found between the Beck Depression Inventory-II (BDI-II) score and RSFC from MFG to hippocampus in ScD group.

**Conclusion:**

Spontaneous neural activities in the right precuneus, left MFG, SFG, and hippocampus were altered in ScD subjects. Functional alterations in these dorsolateral prefrontal cortex and default mode network regions are largely related to abnormal emotional processing in ScD, and indicate strong associations with brain impairments in MDD, which provide insight into potential pathophysiology mechanisms of subclinical depression.

## Introduction

Subclinical depression (ScD) is regarded as an early stage or precursor of major depressive disorder (MDD), because people with ScD experience depressive symptoms that are not severe or persistent enough to merit a diagnosis of MDD (i.e., persistent depressed mood and a series of cognition and physical problems) [1, 2]. According to several surveys in recent years [3], ScD has become highly prevalent among college students worldwide. It was reported that ScD affects 32% of Chinese college students [4] and 23–39% of European college students [5]. ScD is an important factor in suicide [6]. Moreover, a longitudinal study demonstrated that individuals with ScD showed a five-fold increase in their risk of experiencing a first lifetime MDD episode compared to healthy controls [7]. Therefore, it is of great importance to clarify the impairments in neural activity in ScD individuals, as this will provide insight into understanding its biological pathogenesis and prevent the development to MDD.

Among several modalities used to detect brain abnormalities, resting-state functional magnetic resonance imaging (rs-fMRI) can reveal underlying biological mechanisms of mental illnesses by investigating neural activity. Spontaneous low-frequency oscillations (LFOs) of rs-fMRI (0.01–0.08 Hz) are a proven neurophysiologic measure that is highly associated with spontaneous neural activity [8]. Several measures have been proposed to analyze LFOs, such as the power spectrum [9], fluctuation amplitude [10], low-frequency spectral amplitude [11], and amplitude of low-frequency fluctuation (ALFF) [12]. In particular, ALFF measures the square root of the power spectrum in a low-frequency range (absolute strength or intensity of LFOs), representing the spontaneous neural activity at resting state, which has been proven to be an effective and reliable parameter for detecting abnormal neural activity in several mental illnesses [13–16].

Regional homogeneity (ReHo) has also been proven to be a reliable measure for detecting spontaneous neural activity [17]. In this method, it is assumed that the (blood oxygen level dependent) BOLD signal in a voxel is temporally similar to its neighbors, and Kendall’s coefficient concordance (KCC) is used to measure the similarity between them. Thus, ReHo represents the spontaneous neural activity at resting state by calculating the KCC value. In addition, resting-state functional connectivity (RSFC) is further analyzed based on brain regions with altered ALFF and ReHo. RSFC is able to measure the correlations between brain regions by calculating Pearson correlation coefficient. Exploring ALFF, ReHo, and RSFC alterations is of great significance to clarify the functional impairments in MDD and ScD.

Previous studies have reported that fMRI studies are facing the problem of high false positive risk and reproducibility, and emphasized the importance of multiple comparison corrections methods in improving the reproducibility of fMRI studies [18–20]. Different multiple comparison strategies could lead to discrepant results, which has been proved in previous [20] and the current study. But the question is that we cannot determine which correction method is optimal and which result is the most accurate with optimal false positive rate in a limited sample size. To solve this problem, a meta-analysis may help distinguish false-positive from consistent results. Another strategy is to use rigorous and valid statistical methods to analyze fMRI results. Therefore, three kinds of frequently used multiple comparison methods, including the permutation test (PT) [18, 21] with threshold-free cluster enhancement (TFCE) [22], as recommended by Chen, along with the commonly used Gaussian random field (GRF) [23, 24] and false discovery rate (FDR), [25] were applied in this study respectively, so as to avoid the occasional finding caused by single correction strategy. And the common results under different correction methods could be more reliable and convincing.

At present, resting-state functional activity involved in ScD is still unclear, but a growing body of evidence has shown abnormalities in spontaneous brain function in MDD, which is seen as a more serious stage of ScD. Meta-analysis of rs-fMRI in MDD showed altered functional activity in a wide range of brain regions in the frontal-parietal network, frontal-limbic network, and default mode network [26, 27]. In addition, studies of functional connectivity, ALFF, and regional homogeneity in MDD also revealed functional abnormalities in regions including the dorsolateral prefrontal cortex [28, 29], middle frontal gyrus [30], thalamus [31, 32], hippocampus [33], precuneus [34–36], and cerebellum [37]. Therefore, abnormal spontaneous functional activity in ScD may also exist among these areas, which will enhance the understanding of initially impaired regions in MDD.

In this study, ALFF, ReHo, and RSFC analyses were performed to explore abnormalities in resting-state functional activity in college students with ScD compared to healthy controls. Multiple statistical methods, including GRF, FDR, and TFCE with PT, were applied to improve the validity of fMRI results.

## Materials and methods

### Participants

Participants were recruited and screened from a total of 1105 college students at Guangzhou Medical University. ScD subjects were screened using the Beck Depression Inventory-II (BDI-II) [38] scale, which is the gold standard for self-rating nonclinical evaluation of depression. A total of 34 ScD individuals with BDI-II scores of > 10 (11 males, 23 females) and 40 sex-, age-, and education-matched healthy controls with BDI-II scores of < 5 (21 males, 19 females) were enrolled in this study. None of the participants met the diagnostic criteria for MDD according to the Diagnostic and Statistical Manual of Mental Disorders-IV (DSM-IV) [1]. All methods were performed in accordance with DSM-IV. All participants were required to fulfill the following criteria: right-handedness, no visualized lesions in MRI scans, no neurological illness, and no alcohol or drug dependence.

All participants provided written, informed consent after a detailed description of the research, which was approved by the Medical Ethics Committee in the affiliated Guangzhou First People’s Hospital of Guangzhou Medical University.

### MRI data acquisition

All MRI images were acquired using a 3-Tesla MRI scanner (Siemens, Erlangen, Germany) equipped with an 8-channel phased-array brain coil. To minimize head movement and reduce MRI noise, foam pads and headphones were used.

Resting-state fMRI images were obtained using an echo planar imaging (EPI) sequence with the following parameters: echo time (TE) = 21 ms, repetition time (TR) = 2500 ms, flip angle (FA) = 90°, field of view (FOV) = 200 mm × 200 mm, matrix = 64 × 64, voxel size = 3.5 mm × 3.1 mm × 3.1 mm, and 40 slices with no gap. A total of 200 timepoints were scanned. High resolution T1-weighted images were obtained using magnetization prepared rapid acquisition gradient echo (MPRAGE) sequence. Parameters were as follows: TR = 2530 ms, TE = 2.34 ms, FA = 7°, FOV = 256 mm × 224 mm, slice thickness = 1.0 mm with no gap.

During the fMRI scanning process, participants were required to not actively think, keep their eyes closed and relaxed, and stay awake. All images were visually inspected by two experienced radiologists to ensure that no lesions or artifacts were present.

### fMRI preprocessing

Resting-state fMRI data preprocessing was carried out using Data Processing Assistant for Resting-State fMRI (DPARSF, http://www.restfmri.net) [39], which was based on SPM 8 (http://www.fil.ion.ucl.ac.uk/spm/software/spm8) on the MATLAB platform. For each subject, the first 10 volumes were discarded to decrease instability factors from MRI acquisition circumstances and subject habituation. Slice timing correction and realignment were further conducted to compensate for head motion artifacts due to breathing, heartbeats, and uncontrolled slight motion during the scan. Subjects with excessive head motion (2 mm translation or 2° rotation) were excluded. MPRAGE structural images were then registered into the Montreal Neurological Institute (MNI) space with a unified segmentation DARTEL algorithm [40]. Nuisance covariates regression with the Friston 24-parameter model (i.e., six head motion parameters, six head motion parameters one timepoint prior, and the 12 corresponding squared items) was also conducted to filter out head motion effects [41–43]. Next, registration of functional images to the MNI space was performed using structural normalization parameters, and normalization was completed with a resample voxel size of 3 mm × 3 mm × 3 mm. Subsequently, it should be noted that before ALFF calculation, fMRI images were spatially smoothed with full-width at half maximum (FWHM) 6 mm × 6 mm × 6 mm Gaussian kernel [44]. Before ReHo calculation, fMRI images were temporally band-pass filtered (0.01–0.08 Hz) to reduce low frequency drift and physiological high frequency respiratory and cardiac noise [8, 45], and we didn’t perform spatial smoothness before ReHo calculation.

### ALFF and ReHo calculations

ALFF and ReHo were calculated using the Resting-State fMRI Data Analysis Toolkit (REST) package (http://resting-fmri.sourceforge.net) [46]. Briefly, for the ALFF calculation, the time series without the band-pass filter for each voxel was converted to a frequency domain using a Fast Fourier Transform (FFT). Then, the square root at each frequency of the power spectrum was calculated. Because amplitude is proportional to power at a given frequency, amplitude was obtained by calculating the square root of the power spectrum obtained by FFT, and the average squared root was termed the ALFF [12]. ALFF measures the absolute strength or intensity of spontaneous low frequency oscillations. For each subject, ALFF was calculated at each voxel under a frequency of between 0.01 Hz and 0.08 Hz. ALFF maps were then normalized by dividing the ALFF of each voxel by the average ALFF of the whole brain in order to minimize the individual variability. Moreover, ReHo analysis was performed for each subject by calculating the KCC, which was used to measure similarity of the time series of a given voxel to its adjacent 26 voxels. The formula to calculate KCC has been used in previous studies [17]. ReHo maps were then normalized by dividing the KCC of each voxel by the average KCC of the whole brain in order to reduce the influence of individual variations among KCC values.

### Resting-state functional connectivity analysis

Once the clusters with significant group differences in ALFF and ReHo were identified, each cluster would be saved as a region-of-interest (ROI) mask for further RSFC analysis using the seed-based approach. Average time series in each ROI was extracted, and Pearson correlation coefficient was calculated between each two ROIs as the measure of RSFC.

### Statistical analysis

Demographic data from ScD and HC groups were compared using the Statistical Package for the Social Sciences software, version 17 (SPSS, Chicago, IL). Two-sample *t*-tests were performed to assess differences in age and education, and a chi-squared test was performed to assess differences in gender. Comparisons of ALFF and ReHo values between ScD and HC groups were conducted in voxel-based two-sample *t*-tests with the DPABI [47] toolbox. ROI-based FC values between SD and HC groups were compared by two-sample t-test without correction.

It should be noted that several kinds of multiple comparison correction strategies on statistical maps of ALFF and ReHo were carried out to increase test-retest reliability. The first strategy was a two-tailed GRF correlation with a single-voxel threshold of *p* < 0.001 and cluster-defining threshold of *p* < 0.05 [45, 48, 49]. The second method was an FDR correction (*p* < 0.05) [50, 51]. The third strategy was a PT containing 5000 permutations with TFCE, which has been reported to reach the optimal balance between family-wise error rate (< 5%) and test-retest reliability and showed optimal results in recent fMRI studies [20, 52, 53]. Age was also included as a covariate factor in the statistical analysis, with a previous study indicating an association between age and functional activity [54].

Furthermore, linear correlation analyses were performed to identify the relationship between clinical BDI-II scores and altered functional activity and connectivity indexes. Mean ALFF, mean ReHo in each cluster with group difference, and altered RSFC values were extracted firstly. Then, Pearson correlation was used to measure the linear correlations between altered ALFF, ReHo, RSFC and BDI-II scores.

## Results

### Demographic data comparisons

A total of 34 ScD subjects and 40 HC subjects were subjected to fMRI scans. Eight ScD and seven HC subjects were excluded because of excessive head motion (2 mm translation or 2° rotation); thus, the results include 26 ScD and 33 HC subjects. Demographic data are shown in Table 1. There were no significant differences between ScD and HC subjects based on gender, age, and years of education. BDI-II scores in ScD subjects were significantly higher compared to HC subjects (*p* < 0.05).

**Table 1:** Demographic Characteristics of Participants

Characteristics	ScD (*n* = 26)	HC (*n* = 33)	*p*-value
Gender (male/female)	10/16	16/17	0.152^a^
Age (years)	19.69 ± 1.73	19.18 ± 0.87	0.593^b^
Education (years)	13.36 ± 0.92	13.18 ± 0.87	0.492^b^
BDI-II score	22.08 ± 7.34	1.76 ± 1.79	0.000^b^

*Abbreviations*: *ScD* subclinical depression, *HC* healthy control, *BDI-II* Beck Depression Inventory-II. ^a^ and ^b^ indicate the *p*-value for the chi-squared test and two-sample *t*-test, respectively

### Group differences in ALFF

**Fig. 1:**
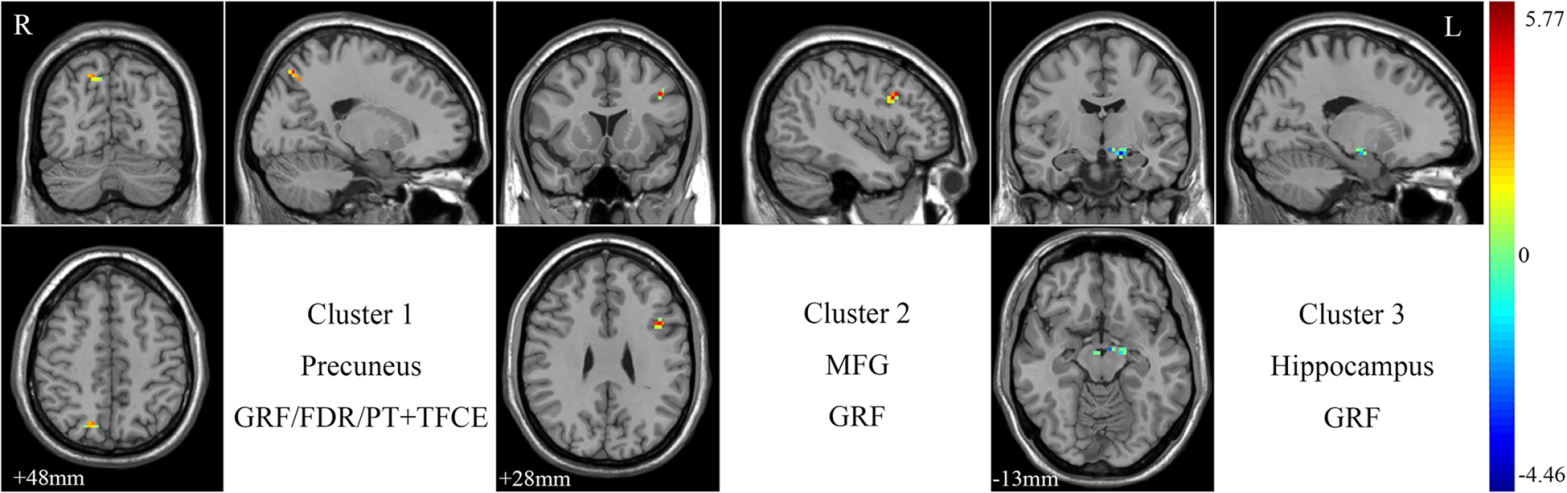
Brain Regions with ALFF Differences Between ScD and HC Subjects. Color bars represent the *t*-value from group analysis. Abbreviations: R, right; L, left; MFG, middle frontal gyrus; GRF, Gaussian random field; FDR, false discovery rate

After performing a two-sample *t*-test with a two-tailed GRF correction, three clusters showed significantly different ALFF values in the ScD group. Specifically, compared to the HC group, the ScD group showed decreased ALFF values in the left hippocampus and increased ALFF values in the left middle frontal gyrus (MFG) and right precuneus, as shown in Fig. 1 and Table 2. After performing an FDR correlation and strict PT + TFCE correction, both results indicated that only one cluster, in the right precuneus, showed significantly increased ALFF values in the ScD group compared to the HC group, as shown in Fig. 1 and Table 2.

**Table 2:** Brain Regions with ALFF Differences Between ScD and HC Subjects

Brain region	Side	Correction	Size (mm^3^)	MNI coordinate (cluster maxima)			*t*-value
				x	y	z	
Precuneus	R	PT + TFCE	14	15	−66	45	5.7677
		GRF	22	15	−66	45	5.7677
		FDR	7	15	−66	45	5.7677
Middle Frontal Gyrus	L	GRF	14	−42	15	33	5.2052
Hippocampus	L	GRF	20	−15	−12	−15	−4.4594

*Abbreviations*: *ScD* subclinical depression, *HC* healthy control, *MNI* Montreal Neurological Institute, *t-value* t statistical value of peak voxel showing ALFF differences between ScD and HC subjects (positive values: ScD > HC; negative values: ScD < HC), *R* right, *L* left, *PT* permutation test, *TFCE* threshold-free cluster enhancement, *GRF* Gaussian random field, *FDR* false discovery rate

Additional exploratory analysis showed no significant correlations (*p* > 0.05) between BDI-II scores and mean ALFF values in these three clusters in the ScD group (precuneus: *r* = 0.066, *p* = 0.749; middle frontal gyrus: *r* = − 0.094, *p* = 0.649; hippocampus: *r* = − 0.229, *p* = 0.260).

### Group differences in ReHo

**Fig. 2:**
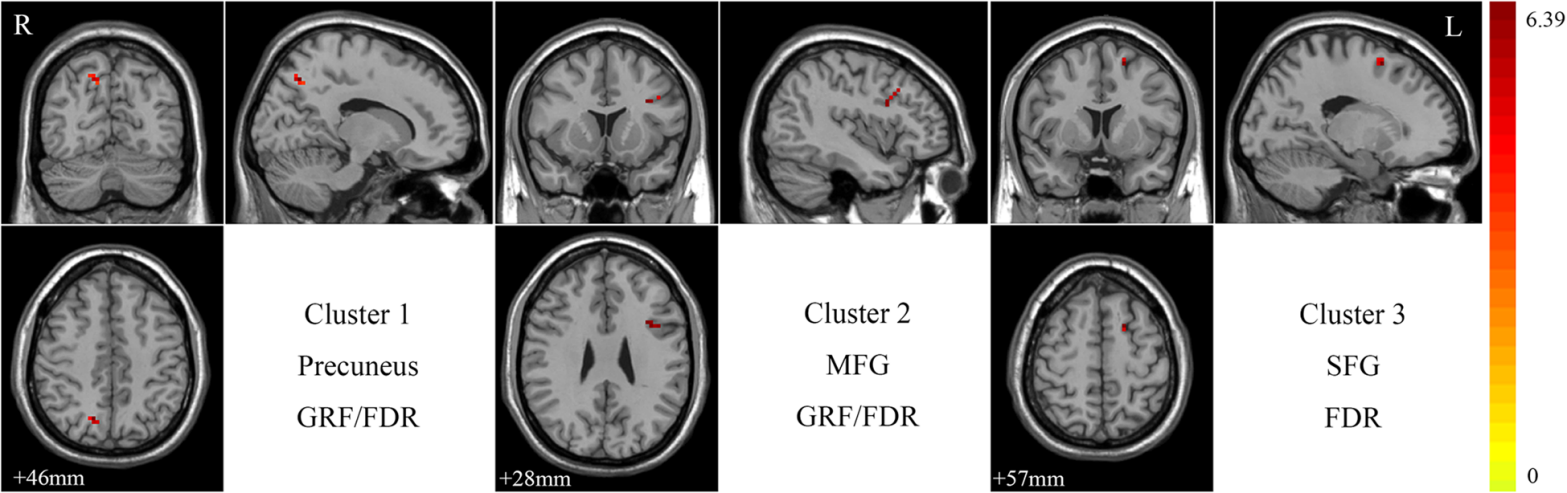
Brain Regions with ReHo Differences between ScD and HC Subjects. Color bars represent the *t*-value from group analysis. Abbreviations: R, right; L, left; MFG, middle frontal gyrus; SFG, superior frontal gyrus; GRF, Gaussian random field; FDR, false discovery rate

After performing a two-sample *t*-test with a two-tailed GRF correction, two clusters showed significantly different ReHo values in the ScD group. Specifically, compared to the HC group, the ScD group showed increased ReHo values in the left MFG and right precuneus. After performing an FDR correlation, three clusters showed significantly increased ReHo values in the left MFG, right precuneus, and left superior frontal gyrus (SFG). No cluster was found under a PT + TFCE correction. All ReHo results are shown in Fig. 2 and Table 3.

**Table 3:** Brain Regions with ReHo Differences Between ScD and HC Subjects

Brain region	Side	Correction	Size (mm^3^)	MNI coordinate (cluster maxima)			*t*-value
				x	y	z	
Precuneus	R	GRF	11	15	−66	45	5.8517
		FDR	3	16	−66	46	5.8517
Middle Frontal Gyrus	L	GRF	11	−41	19	36	4.1707
		FDR	3	−36	13	47	5.5239
Superior Frontal Gyrus	L	FDR	5	−18	7	57	6.3915

*Abbreviations*: *ScD* subclinical depression, *HC* healthy control, *MNI* Montreal Neurological Institute, *t-value* t statistical value of peak voxel showing ALFF differences between ScD and HC subjects (positive values: ScD > HC; negative values: ScD < HC), *R* right, *L* left, *GRF* Gaussian random field, *FDR* false discovery rate

Additional exploratory analysis showed no significant correlations (*p* > 0.05) between BDI-II scores and mean ReHo values in these three clusters in the ScD group (precuneus: *r* = 0.230, *p* = 0.254; MFG: *r* = − 0.1.67, *p* = 0.416; SFG: *r* = − 0.185, *p* = 0.365).

### Group differences in RSFC

A total of 6 RSFCs among the right precuneus, left hippocampus, left MFG, and left SFG were obtained by calculating the Pearson correlations between time series of each two ROIs. After two-sample t-test between ScD and HC groups, ScD showed increased RSFC values from the left MFG to the left hippocampus (*p* < 0.05). Moreover, significant positive correlation was found between the BDI-II score and RSFC from MFG to hippocampus in ScD group (*r* = 0.5822, *p* = 0.0018).

## Discussion

To the best of our knowledge, this is the first study combining multiple indicators of spontaneous neural activity to explore brain functional impairments in ScD. A major strength of the study is its use of multiple strict comparisons (i.e., GRF, FDR, and PT + TFCE) to ensure repeatability and validation of the results. The right precuneus and left MFG showed the most significant functional abnormalities based on both ALFF and ReHo results with strict statistical strategy. Increased ReHo values were observed in the left SFG, and decreased ALFF values were observed in the right hippocampus with relatively less statistical power.

The precuneus showed increased ALFF and ReHo values in ScD subjects with the strictest statistical strategy, which should be considered notable. Precuneus abnormalities have been frequently reported in recent structural and functional MRI studies of MDD. According to the structural abnormalities, volume [55], white matter fiber connectivity to the anterior cingulate gyrus (ACC) [56], and cortical gyrification [57] of the precuneus were reduced in previous studies. Moreover, after investigating functional abnormalities, spontaneous neural activity of the precuneus was found to be impaired in MDD patients as measured by ALFF and ReHo [58–60]. Functional connectivity of the precuneus with the dorsolateral prefrontal cortex, MFG, ACC, hippocampus, and other regions were found to be altered at the voxel level or large-scale ROI level [27, 35, 61–63]. Therefore, structural and functional abnormalities of the precuneus are greatly associated with depression.

The precuneus has been rarely reported in previous MRI studies of ScD. One study showed that gray and white matter volumes of the precuneus were increased in young women with ScD [64]. Another study showed that functional connectivity of the default mode network (DMN), including the precuneus, was reduced [65]. The present research conducted ALFF and ReHo analyses and confirmed that spontaneous functional activity of the precuneus was altered in ScD. That is to say, initial impairment of the precuneus in MDD may start during an early stage of ScD. Furthermore, the precuneus is a medial parietal region greatly associated with spatial function, navigation, and memory [66], and it plays a core role in DMN activity and cognitive processing [67]. So, it is hypothesized that increased spontaneous neural activity in the right precuneus in ScD may cause cognition and sensory dysfunction. Furthermore, it may lead to larger scale alternations in the DMN in ScD and MDD, preceding the development of depressive episodes.

The left hippocampus showed alternations only based on ALFF values after GRF correction, and the degree of abnormality may be lower than precuneus and MFG. Brain structural and functional impairments of the left hippocampus have been frequently reported in previous studies. Gray matter volume has been demonstrated decreased in MDD based on a meta-analysis [68]. Functional connections of the hippocampus with the MFG, inferior parietal cortex, and middle temporal gyrus were found to be impaired in MDD [27, 33]. Moreover, after investigating functional abnormalities in ScD, aberrant functional connections from the hippocampus to SFG, MFG and cuneus have also been reported in older adults with ScD [69]. The presented results further support the functional impairments in the hippocampus and increased functional connection from hippocampus to MFG in younger college students with ScD. The hippocampus is an important subcortical region involved in the processing of memory, perception, and emotional regulation [70]. Therefore, alternations of functional activity in the left hippocampus may be largely associated with depression symptoms and cause the depressive mood and cognition dysfunction.

It is worth noting that both the right precuneus and left hippocampus are important regions in DMN, the functional impairments of which have been confirmed in previous studies [27]. Altered spontaneous neural activities in the right precuneus and left hippocampus indicate that DMN dysfunction may start during early subclinical stage of depression. It is known that depression symptoms are associated with excessive self-focus, a tendency to engage oneself in self-referential processing [71]. DMN is responsible to spontaneous cognition, self-referential processing, and emotional regulation [72]. After taking this evidence into consideration, it is hypothesized aberrant DMN function may lead to self-referential processing abnormally integrating with biased emotional memory in ScD.

In the frontal lobe, both of the left MFG (especially in the inferior frontal junction) and the left SFG indicate increased spontaneous neural activity in ScD. MFG is involved in regulating the strength of reactions to emotional stimuli, and alterations in functional activity of the MFG may cause inappropriate responses to emotional events. The SFG is generally considered a core brain region in the cognitive control system [73] and emotional regulation-related processes, especially regarding feelings of amusement [74]. These are influential factors for depressive symptoms.

It should be noted that the MFG and SFG are crucial parts of the dorsolateral prefrontal cortex (DLPFC), which has been tightly linked to depression. Higher functional activity in the DLPFC has been frequently observed in fMRI studies of MDD [27], and hyperactivity of the brain region is correlated with depression severity [75]. In addition, the DLPFC also serves as a primary target region for depression treatment using repetitive transcranial magnetic stimulation (rTMS) [76]. The presented ALFF and ReHo results further demonstrate that dysfunction in the DLPFC may start during early subclinical stage of depression. The DLPFC is believed to be critical for attention regulation and emotional judgment, and it is speculated that functional impairments in the DLPFC have a direct influence on the inappropriate responses to emotional events seen in individuals with ScD.

We also found a significantly positive correlation between depression severity and the RSFC of hippocampus-MFG. Both hippocampus and MFG are associated with emotion regulation [70, 77], and they are essential parts of the limbic-cortical circuit, which is a network consisting of the frontal cortex, anterior cingulate cortex, hippocampus, and anterior thalamus. In this limbic–cortical model, abnormal activity in limbic areas (including the hippocampus) is not adequately controlled by prefrontal areas, with an associated depressed mood [78]. Limbic–cortical model plays a critical role in the pathophysiology of MDD. Increased RSFC of hippocampus-MFG and its positive correlation with depression severity were found in ScD, which might further prove that the dysfunction of the limbic-cortical circuit serves as a key factor leading to depression.

We found that the voxels size of FDR corrected ReHo results is relatively small, which might limits its persuasion. The principle of FDR is different from family-wise error correlation (including GRF and TFCE+PT) in that FDR doesn’t take the relationships among voxels into consideration and it is relatively more strict in this study, so the number of voxels based on FDR is relatively less than GRF and TFCE+PT. In addition, it should be noted that a strong consistency could be found among the results of FDR GRF and TFCE+PT, improving the reliability of the results, especially in the right precuneu and left MFG.

Several limitations of this study should be noted. First, although subjects were screened from more than 1000 college students, the sample size was still relatively small, which limits the statistical power of this study. Thus, a larger sample size and multiple cohorts of individuals with ScD are warranted in future studies. Second, according to statistical methods, there was no conclusion that included the optimal correction strategy among the FDR, GRF, and PT + TFCE. The statistical method is not only a concern of this study but also of most fMRI studies, because it plays a decisive role in the reliability of the results. Therefore, more efforts on exploring the statistical methods in fMRI research are greatly encouraged in future studies.

## Conclusion

In summary, this study revealed altered spontaneous neural activity in the right precuneus, left MFG, SFG, and hippocampus in ScD subjects using valid statistical methods. These dorsolateral prefrontal cortex and default mode network regions are largely related to abnormal emotional processing in ScD, and indicate strong associations with brain impairments in MDD. These findings provide insight into potential pathophysiology mechanisms of subclinical depression.

## Data Availability

The datasets used or analysed during the current study are available from the corresponding author on reasonable request.

## Abbreviations

ALFF: Amplitude of low-frequency fluctuation
ACC: Anterior cingulate gyrus
BDI-II: Beck Depression Inventory-II
BOLD: Blood oxygen level-de-pendent
DPARSF: Data processing assistant for resting-state fMRI
DMN: Default mode network
DSM-IV: Diagnostic and statistical manual of mental disorders-IV
DLPFC: Dorsolateral prefrontal cortex
EPI: Echo planar imaging
TE: Echo time
FDR: False discovery rate
FFT: Fast Fourier transform
FOV: Field of view
FA: Flip angle
FWHM: Full-width at half maximum
GRF: Gaussian random field
HC: Healthy control
KCC: Kendall’s coefficient concordance
LFOs: Low-frequency oscillations
MPRAGE: Magnetization prepared rapid acquisition gradient echo
MDD: Major depressive disorder
MFG: Middle frontal gyrus
MNI: Montreal neurological institute
PT: Permutation test
REST: Resting-state fMRI data analysis toolkit
ReHo: Regional homogeneity
TR: Repetition time
rTMS: Repetitive transcranial magnetic stimulation
rs-fMRI: Resting-state functional magnetic resonance imaging
SPSS: Statistical package for the social sciences
ScD: Subclinical depression
SFG: Superior frontal gyrus
TFCE: Threshold-free cluster enhancement
